# Rosiglitazone amplifies the sensitivity of docetaxel and reduces the expression of CD44v6

**DOI:** 10.3892/ol.2014.1824

**Published:** 2014-01-24

**Authors:** FAHE JI, DONGCHU MA, ZHAOZHE LIU, XIAODONG XIE

**Affiliations:** Department of Oncology, The General Hospital of Shenyang Military Command, Shenyang, Liaoning 110840, P.R. China

**Keywords:** docetaxel, rosiglitazone, cell proliferation, cell cycle, CD44v6

## Abstract

Breast cancer seriously impairs physical and mental health in females. Currently, with further investigation into drugs, a number of new pharmacological effects have been found that offer new methods for clinical application in the treatment of breast cancer. As a widely used antidiabetic drug, rosiglitazone (Ros) has become well known for its anticancer effects, mediated by the activation of peroxisome proliferator-activated receptor γ and downregulated expression of the associated invasion gene. The objective of the present study was to investigate the combination of Ros and docetaxel (DOC) and whether DOC has any effect on breast cancer cell lines. The results showed that the combination of Ros and DOC may cooperate to increase anti-growth efficacy. The additive inhibitory effects on cell proliferation were sequence-dependent and are not likely to be associated with cell cycle arrest. This suggested that the target activation of associated factors of the signaling pathway by Ros may be a compelling ally in cancer treatment. In addition, evidence was provided for a convergence of Ros and DOC to induce the reduced expression of CD44v6. Future studies are required to confirm which associated gene of Ros is significant in blocking the signaling pathway.

## Introduction

Breast cancer is one of the most common types of female malignant tumor and its incidence continues to exhibit an upward trend year-by-year. As developments have been made concerning the introductory biological behavior of breast cancer and clinical studies, the level of the overall treatment of breast cancer has made significant progress. Peroxisome proliferator-activated receptors (PPARs) are members of the nuclear hormone receptor super family and rely on nuclear ligands for the activation of transcription. PPARs exhibit certain effects on adipose formation, lipid metabolism, maintaining blood glucose stability and modulation of the inflammatory process. In previous years, the role of PPARγ in tumorigenesis has attracted particular attention. Previous studies have found that PPARγ, as an important cellular regulatory factor, is not only expressed in normal adipose tissue and the immune system, but is also highly expressed in colon, pancreatic, lung and other types of cancer (1–3). PPARγ inhibits cell proliferation and tumor angiogenesis, induces apoptosis and reduces invasiveness following activation by specific ligands, such as the prostaglandin D2 metabolite, 15-d-PGJ2 and rosiglitazone (Ros).

Cell adhesion molecule CD44 is an important member of the intercellular adhesion molecule family and is widely spread in white blood cells and epithelial and endothelial cells. CD44 mainly mediates the adhesion of cells to the matrix. The variant exon transcript of CD44 is known as CD44v, which is mainly expressed in pathological processes. Previous studies have shown that CD44v6 may enhance the invasion and metastasis of tumor cells by changing the composition and function of cell surface adhesion molecules.

At present, docetaxel (DOC) is used for various types of cancer, including breast, non-small cell lung and gastric cancer, with satisfactory results (4–6). Previous clinical results have shown that conventional high-dose chemotherapy in patients may not only result in different degrees of toxicity, but also lead to irreversible damage and even endanger the lives of patients. A previous study has demonstrated that Ros synergizes the anticancer activity of cisplatin, reduces the nephrotoxicity of cisplatin, increases patient sensitivity to chemotherapy and reduces the toxicity of chemotherapy through dose reduction (7). Further clarification of the mechanism of tumor invasion and metastasis is likely to be of benefit and simultaneously provide new theories and approaches for cancer prevention and drug treatment. The present study investigated the association of Ros and DOC with the inhibition and micrometastasis to MCF-7.

## Materials and methods

### Cell culture

The human breast cancer MCF-7 cell line was obtained from the Institute of Biochemistry and Cell Biology, (Chinese Academy of Sciences; Shanghai, China). MCF-7 cells were cultured in RPMI-1640 (Hyclone, Waltham, MA, USA) containing 10% fetal bovine serum (Tianjin Hao Yang Biological Products Co., Ltd., Tianjin, China) with the addition of antibiotics. The cell line was maintained at 37°C in a humid atmosphere and 5% CO_2_.

### Cell viability assay

Cells were seeded at a density of 5×10^3^ cells/well in 96-well plates containing 100 μl complete medium in quintuplicate. The cells were allowed to attach overnight prior to treatment with the indicated doses of Ros (Shanghai Sunve Pharmaceutical Co., Ltd., Shanghai, China) and DOC (Qilu Pharmaceutical Co., Ltd., Jinan, China) for 24, 48 and 72 h. Subsequently, viable cells were treated with 5 mg/l 3-(4,5-dimethylthiazol-2-yl)-2,5-diphenyltetrazolium bromide (MTT; Nanjing KeyGEN Biotech Co., Ltd., Nanjing, China) for 4 h and MTT-formazan conversion was analyzed by automated ELISA reader at 540 nm. The formula was used to calculate inhibition rate was: $\text{Inhibition rate }(\%)=[(\text{control group }{\text{A}}_{540}-\text{sample group }{\text{A}}_{540})/\text{control group }{\text{A}}_{540}]\times 100$. In addition, the combined effect of the two drugs was calculated using the formula: $\text{q}={\text{E}}_{\left(\text{A}+\text{B}\right)}/[{\text{E}}_{\text{A}}+(1\;{\text{E}}_{\text{A}})\times {\text{E}}_{\text{B}}]$.

### Cell cycle analysis

Cells were trypsinized, washed in phosphate-buffered saline (PBS; Hyclone) and centrifuged at 200 × g. Pellets were then fixed in 5 ml ethanol (70%) and stored at −20°C until use. Cells were centrifuged and pellets resuspended in 200 μl PBS and 10 mg/l RNAse A (Sigma-Aldrich, St. Louis, MO, USA)was incubated for 30 min at 37°C. Subsequently, the cells were resuspended in propidium iodide solution [0.1% sodium citrate, 0.1% Triton X-100 (Sigma-Aldrich) and 50 μg/ml propidium iodide (Sigma-Aldrich)]. Cell cycle analysis was performed by flow cytometry (BD FACScalibur; BD Biosciences, Franklin Lakes, NJ, USA). Data were analyzed using ModFit LT software (Verity Software House, Topsham, ME, USA).

### Reverse-transcription polymerase chain reaction (RT-PCR)

Treatment with various drugs was added to logarithmic growth phase cells. The RNA from cells was extracted using the total RNA extraction kit [centrifugal columnar type; Tiangen Biotech (Beijing) Co., Ltd., Beijing, China]. The RNA was reverse transcribed into complementary DNA (cDNA) according to the manufacturer’s instructions. PCR primers were purchased from Takara Bio, Inc. (Shiga, Japan). The primers used for PCR amplification were: sense, 5′-AGACAGAAATGGCACCAC-3′ and antisense, 5′-AATGGGAGTCTTCTTTGG-3′ for CD44v6 (224-bp product); and sense, 5′-AATCCCATCACCATCTTCC-3′ and antisense, 5′-CATCACGCCACAGTTTCC-3′ for GAPDH (382-bp product). Reaction system of PCR (20 μl altogether) included 2 μl cDNA, 1 μl forward primer (10 μmol/l), 1 μl reverse primer (10 μmol/l), 25μl 2X TransTaq™ HiFi PCR SuperMix II and 21 μl ddH_2_O. The conditions for PCR were as follows: predenaturation at 94°C for 5 min, 94°C for 30 sec, 50°C for 30 sec and 72°C for 30 sec, for 36 cycles; and elongation at 72°C for 5 min. PCR products were detected by 2% agarose gel. The band density was detected using the Bio-Rad Quantity One software (Bio-Rad, Hercules, CA, USA). The relative band density represented the relative expression levels of CD44v6 and was calculated using the formula: Relative density of bands=CD44v6 band density/GAPDH band density.

### Statistical analysis

Statistical differences were assessed using SPSS 13.0 software (SPSS, Inc., Chicago, IL, USA). Data are expressed as the mean ± standard deviation of at least three independent experiments. All the groups were studied in parallel and differences between groups were analyzed using analysis of variance (ANOVA), as appropriate. Bonferroni’s post-hoc test was used for multiple non-pairwise comparisons of means. Multiple comparisons among means were performed using the least significant difference t-test. P<0.05 was considered to indicate a statistically significant difference.

## Results

### Ros and DOC inhibit cell proliferation

**Figure 1:**
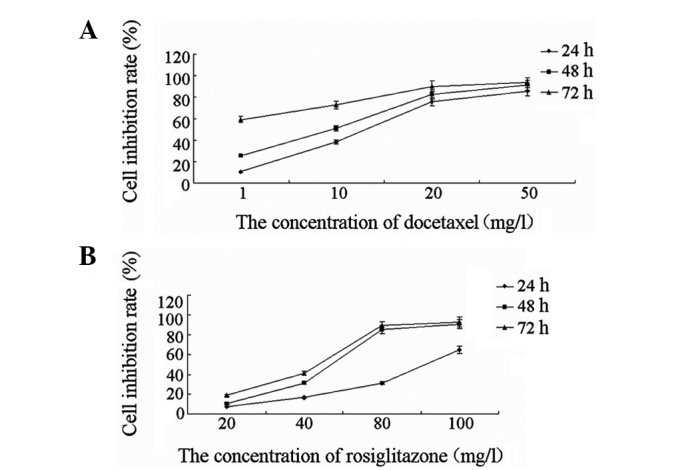
Cell inhibition rate of each group. MCF-7 cell lines with (A) docetaxel and (B) rosiglitazone alone.

To examine the effects of Ros and DOC on cancer cell growth, MCF-7 cell lines were treated with Ros or DOC alone or in combination and cell viability was determined. As shown in Fig. 1, Ros and DOC inhibited cell viability, as compared with the vehicle-treated cells. Each drug inhibited cell proliferation in a time- and dose-dependent manner (P<0.05).

**Figure 2:**
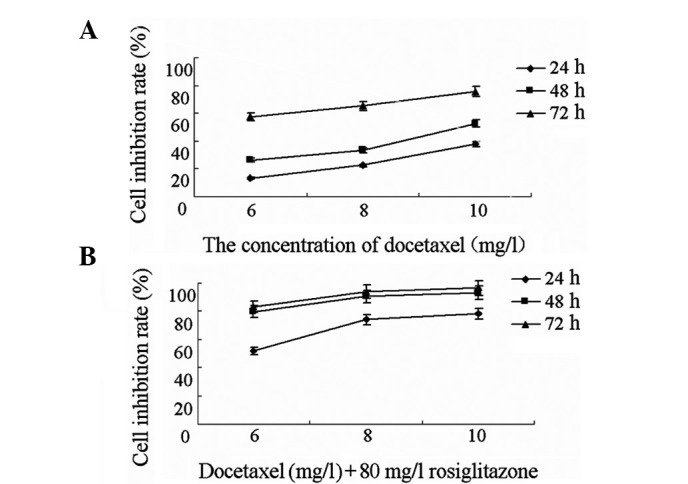
Comparison of cell inhibition rates between individual and combination treatments. MCF-7 cell lines with (A) docetaxel alone and (B) combination of rosiglitazone and docetaxel.

Table I and Fig. 2 show that in the combined treatment, Ros potentiated DOC action on MCF-7 cells. Ros and DOC were also shown to inhibit cell viability, as compared with the vehicle-treated cells. In addition, the combined treatment was more effective than the two treatments alone. Combination of Ros and DOC exhibited additive effects for the growth inhibition of MCF-7 cells in a time- and dose-dependent manner (P<0.05).

### Effect of Ros and DOC on cell cycle

To evaluate the mechanism of growth inhibition by Ros and DOC, the cell cycle profile was analyzed by flow cytometry following treatment with Ros or DOC alone or a combination of the drugs.

**Figure 3:**
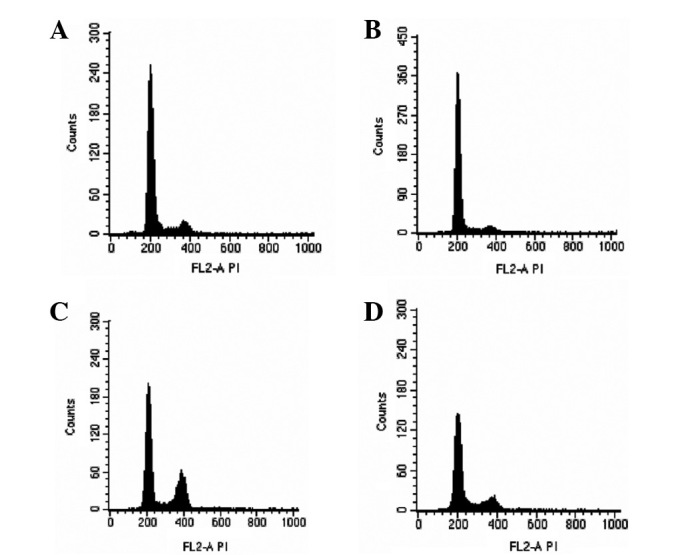
Cell cycle arrest of each group by flow cytometry. Cell cycles of the (A) control, (B) rosiglitazone, (C) docetaxel and (D) combination groups. PI, propidium iodide.

Details of tumorigenicity are shown in Fig. 3. Ros treatment resulted in an increase of cells in G1-phase arrest compared with the control group (85.93±0.70, vs. 75.29±0.28%), with a decrease of cells in the S and G2/M phases. DOC treatment, as predicted, resulted in an increase in the number of cells in the G2/M phase compared with the control group (20.61±1.26, 25.23±1.27, 27.75±1.52, vs. 5.83±0.81%) and a reduction in the number of cells in the S and G1 phases in MCF-7 cell lines.

Ros combined with DOC treatment resulted in a decrease in the number of cells in G1 phase and an increase in cells in the S and G2/M-phase arrest in MCF-7 cell lines, with increasing concentrations of DOC.

### Effect of Ros and DOC treatment on the expression of CD44v6 messenger RNA (mRNA)

**Figure 4:**
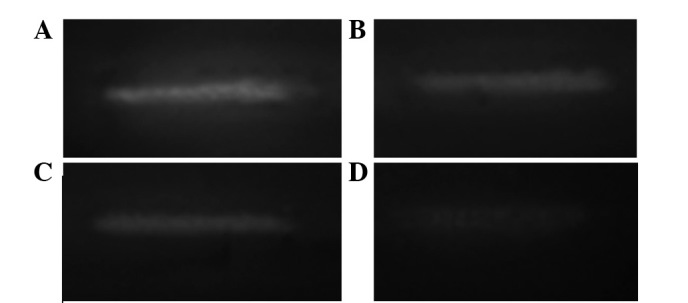
Expression of STAT3 messenger RNA of each group by reverse transcription polymerase chain reaction. (A) Control, (B) 10 mg/l DOC, (C) 80 mg/l Ros and (D) 10 mg/l DOC + 80 mg/l Ros groups. Ros, rosiglitazone; DOC, docetaxel.

To evaluate the effect of associated factors on invasion and micrometastasis by Ros and DOC, the micrometastasis-associated gene, CD44v6, was analyzed by RT-PCR following treatment with Ros or DOC alone or in combination. As shown in Fig. 4 and Table II, DOC treatment caused the downregulated expression of CD44v6 mRNA significantly (relative expression, 0.0196±0.0012) and Ros treatment resulted in a reduction in the expression of CD44v6 mRNA (relative expression, 0.02963±0.001). The combined treatment of Ros and DOC resulted in a significant enhancement effect, downregulating the expression of CD44v6 compared with the two treatments alone (relative expression, 0.0178±0.0008).

## Discussion

DOC is an indispensable chemotherapy drug for the treatment of metastatic breast cancer. Previously, DOC has been found to evidently inhibit the proliferation of MCF-7 cells *in vitro*, with an inhibition rate of 1.21–83.06%, which is consistent with the results of the current study (8). The present study showed that DOC treatment results in an increase in MCF-7 cells in G2/M-phase arrest 24 h later (9) In addition, the functional intensity developed gradually in a dose-dependent manner, which is consistent with previous studies in the literature. Although the results of a previous phase III clinical trial of DOC on the treatment of breast cancer showed that a DOC-based regimen increases survival and reduces the risk of breast cancer recurrence, the experimental results also showed that there are more serious toxicities compared with paclitaxel, including stage III–IV neutrocytopenia, neutropenic fever, diarrhea, languor infection and neurotoxicity, even including three patients with DOC-related mortality (10).

It has been confirmed that low toxicity combination chemotherapy drugs may reduce the toxic effects on the body by reducing the dose of the chemotherapy drug while simultaneously enhancing chemosensitivity (11). As a PPARγ-specific ligand, Ros has relatively few and milder toxic effects. The majority of previous studies have supported that PPARγ is a factor of good prognosis for breast cancer. In addition, studies have shown that ligand-activated PPARγ results in G1-phase arrest and inhibits cell proliferation by affecting the G1/S checkpoint of various tumor cell lines (12). Ros, as an insulin sensitizer, has been used primarily for the clinical treatment of endocrine diseases. The results of the present study suggest that the combined treatment of Ros and DOC may exhibit synergistic and additive effects. The experimental results may be clearer with the sequential treatment of Ros and DOC and may determine the effect on cell cycle by Ros combined with DOC.

The resistance to chemotherapy drugs is the main cause responsible for failure in treating malignant tumors. The possible reasons for multidrug resistance include effective inhibition concentrations, DNA damage repair, gene mutation and dysfunction of signal transduction. The phosphoinositide 3-kinase (PI3K)/Akt/mammalian target of rapamycin (mTOR) signaling pathway is extremely important in the regulation of the cell cycle, tumor cell proliferation, apoptosis, tumor invasion, metastasis, angiogenesis and multidrug resistance (13). Inhibiting the PI3K/Akt/mTOR signaling pathway enhances epirubicin, cisplatin, DOC and other chemotherapy treatments (14–16).

mTOR is a serine/threonine kinase and is one of the downstream effectors of the PI3K/Akt signaling pathway. As a member of the PI3K-related protein kinase family, mTOR may regulate cell growth and the cell cycle and activated PI3K/Akt activates mTOR. Activated mTOR promotes cell access to the S phase from the G1 phase by promoting the expression of a variety of cyclin-dependent kinases (17).

Phosphatase and tensin homolog deleted on chromosome 10 (PTEN) is a suppressor gene with the activity of phosphatase and a negative modulator of the PI3K-Akt pathway. The reason for the loss of messenger function is that phosphatidylinositol (3,4,5)-trisphosphate (PIP3) is hydrolyzed to phosphatidylinositol (4,5)-bisphosphate by the PTEN encoded protein (18). Loss of PTEN function activates downstream signaling transduction pathways excessively through the accumulation of PIP3 (19). Excessive activation of PI3K/Akt/mTOR causes the following variations (20). Firstly, excessive proliferation-activated Akt promotes the G1/S transition by blocking the degradation of cyclin D1. Previous evidence (21) has shown that inhibition of the PI3K/Akt/mTOR pathway causes cells to arrest in the G1 phase. Secondly, when PI3K/Akt/mTOR continues to be activated, tumor cells show antiapoptotic properties in the growth and treatment process. The result is that the increasing expression of wild-type PTEN inhibits the PI3K/Akt pathway and further inhibition of mTOR enhances the virtue of chemotherapy drugs and reversal of multidrug resistance. Previous studies have identified that the activation of PPARγ may inhibit proliferation and reduce the invasiveness of tumor cells by blocking PI3K/Akt by upregulating the expression of PTEN (22).

CD44 is an important member of the adhesion molecule family and is closely associated with tumor invasion and metastasis. CD44 is multifunctional and involved in a number of aspects of invasion and metastasis, including the adhesion interaction between cells and cells to the extracellular matrix. The majority of previous studies have shown that the expression of CD44v6 correlates with breast cancer occurrence, progression, infiltration and micrometastasis. Its high expression may be considered as one of the indices for evaluating the prognosis of breast cancer (23). The results of a previous study by Looi *et al* (24) showed that the lung metastatic potential of high CD44 expression in breast cancer cells was significantly higher than that of the low expression. In addition, previous studies have shown that chemotherapy drugs inhibit the expression of CD44v6 by killing tumor cells (25).

The results of the present study suggest that MCF-7 cells with a low CD44v6 expression were found until 24 h following administration of DOC. An additional study found that cyclooxygenase-2 (COX-2) inhibitors activate the ligands of PPARγ and it has been suggested that the antitumor efficacy of the selective COX-2 inhibitor is associated with upregulated PPARγ expression (26). COX-2 and CD44v6 expression exhibit synergistic effects and COX-2 may promote invasion and metastasis through the upregulated expression of CD44v6 (27,28). Leung *et al* (29) previously confirmed that Ros may inhibit the proliferation of MKN45 cells and COX-2 mRNA expression in a concentration-dependent manner. The present study results showed that Ros downregulates the expression of CD44v6. In addition, statistical significance was identified in the treatment effects of the expression of CD44v6 with Ros and DOC in combination or alone. However, future studies on this mechanism are required.

In conclusion, the PPARγ ligand Ros may enhance the cytostatic effect of DOC on MCF-7 cells. Although the combined treatment of drugs may have an additive effect, the results may show no marked correlation between cell inhibition and cell cycle arrest. The current study provided experimental evidence for Ros and DOC in inhibition and invasion. However, the pathway of the PPARγ ligand in breast cancer is relatively complicated and the PPARγ ligand cross-talks with other signaling pathways. The antitumor activity of Ros combination chemotherapy for breast cancer requires further investigation.

## Figures and Tables

**Table I:** Inhibition rate of cell growth and q-value of each group.

	24 h		48 h		72 h	
			
Groups	Inhibitory rate, %	q-value	Inhibitory rate, %	q-value	Inhibitory rate, %	q-value
6 mg/l DOC + 80 mg/l Ros	51.53±0.56^a^	1.29	79.65±0.78^a^	1.20	83.02±1.21^a^	0.86
8 mg/l DOC + 80 mg/l Ros	74.11±1.89^a^	1.60	90.15±1.72^a^	1.30	93.69±0.32^a^	0.97
10 mg/l DOC + 80 mg/l Ros	77.94±1.98^a^	1.37	93.40±0.66^a^	1.20	96.27±0.22^a^	0.98
6 mg/l DOC	12.92±1.36		26.27±1.09		57.18±0.42	
8 mg/l DOC	22.17±1.31		33.03±1.90		64.95±0.76	
10 mg/l DOC	37.36±2.14		52.34±0.88		75.55±1.06	

a P<0.05, vs. DOC alone.

Results from the 3-(4,5-dimethylthiazol-2-yl)-2,5-diphenyltetrazolium bromide assay showed a significantly reduced proliferation of MCF-7 cells at 24, 48 and 72 h following combination of Ros and DOC compared with DOC alone. q>1.15 indicated a synergistic effect, q=0.85–1.15 indicated an additive effect and q<0.85 indicated a protective effect. Ros, rosiglitazone; DOC, docetaxel.

**Table II:** Relative expression levels of CD44v6 mRNA of each group by reverse transcription polymerase chain reaction.

Groups	Relative expression levels
Control	0.0342±0.0012
10 mg/l DOC	0.0196±0.0012^a^
80 mg/l Ros	0.02963±0.001^a^
10 mg/l DOC + 80 mg/l Ros	0.0178±0.0008^a^,^b^

a P<0.05, vs. control group.

b P<0.05, vs. 10 mg/l DOC group and 80 mg/l Ros group.

Ros, rosiglitazone; DOC, docetaxel.
